# Anxiety severity among adults having upper gastrointestinal symptoms: Proportion and predictors - A cross-sectional study

**DOI:** 10.6026/973206300213567

**Published:** 2025-10-31

**Authors:** Ali Rashida, Solanki Vindhya, F. Sutar Roshan, Sahu Pushpendra, R. Rozatkar Abhijit, Prasad Pankaj, Kumar Sanjeev, Bali Surya, Dabar Deepti, Kumar Mohit, Majumdar Anindo

**Affiliations:** 1 Department of Community & Family Medicine, AIIMS Bhopal, Madhya Pradesh, India; 2 Department of Psychiatry, AIIMS Bhopal, Madhya Pradesh, India; 3 Department of Community & Family Medicine, AIIMS Bhopal, Madhya Pradesh, India and Department of Community Medicine, Government Medical College, Satna, Madhya Pradesh India

**Keywords:** GAD-7, Gastrointestinal disorders, anxiety, screening, Upper GI disorders

## Abstract

Anxiety is common in upper GI disorders and yet data from India is lacking. Therefore, it is of interest to document the proportion and predictors of severity of anxiety in adults with upper GI symptoms. In this cross-sectional study from a tertiary hospital in Bhopal, India, exit interviews of patients >18 years age, with upper GI symptoms were conducted using semi-structured tools. Out of 500 participants, 16.6% had minimal, 16% mild, 53.4% moderate 14% had severe anxiety as per GAD-7 criteria. Being aged 65 and above, married, employed, vegetarian, high likelihood of GERD were predictors, whereas, higher socio-economic status and alcohol consumption were protective against moderate to severe anxiety. Collaborative care models focused on targeted screening for anxiety among these patients is need of the hour.

## Background:

Upper gastrointestinal (GI) symptoms refer to a wide range of symptoms, including dyspeptic symptoms as well as gastro-esophageal reflux symptoms. A systematic review reported that the prevalence of upper abdominal pain or discomfort ranged from 8-54% [1]. Functional gastro-intestinal disorders are one of the commonest digestive diseases worldwide leading to significant morbidity and thus burden on healthcare resources [2]. Gastroesophageal reflux disease (GERD) is one of the most common, recurrent and comorbid gastroenterological diseases, with a prevalence of 10% in East Asia in the adult population [3]. The prevalence of GERD ranged from 5% to 28.5% [4]. It has been observed that in general practice settings, around 4% of consultations are patients suffering from upper gastrointestinal complaints [5]. Generalized anxiety disorder (GAD) is the most frequent anxiety disorder in primary care, is present in 22% of primary care patients complaining of anxiety problems and thus, GAD patients are high users of primary care resources [6]. Previous literature reports that anxiety is associated with gastrointestinal symptom burden, sleep disturbances and decreased health-related quality of life (HRQoL), especially in patients with functional bowel disorders [7, 8, 9- 10]. It has been reported that psychological disorders, such as anxiety and depressive disorders, often precede or exacerbate functional gastrointestinal disorder (FGID) symptoms and correlate with symptom severity and health outcomes [11]. Therefore, it is of interest to study of anxiety severity among adults having upper gastrointestinal symptoms.

## Methods:

The present cross-sectional study was conducted in the general outpatient clinic of a tertiary care teaching hospital in Bhopal, India. Ethical approval was obtained from the Institutional Human Ethics Committee (IHEC) vide Ref no. IHEC-LOP/2019/IM0213. Written Informed consent was obtained from all the participants before enrollment in the study. This analysis is part of an umbrella project aimed at finding out the proportion and predictors of anxiety, depression and sleep quality. Data was collected from November 2019 to February 2023 from the clinic, which had functioned between 2012 to 2023, with intermittent halt of services during the COVID-19 pandemic. Patients >18 years reporting with symptoms of upper gastrointestinal (GI) disorders and those willing to participate were included. Patients having established (diagnosed/on treatment) hypertension, diabetes, chronic respiratory diseases, cardiovascular disease, moderate to severe anemia, thyroid disorders and any established neurological condition were excluded. For operationally defining symptoms of upper gastrointestinal disorders, the International Foundation for Gastrointestinal Disorders (IFFGD) definition was followed [12]. The symptoms considered were heartburn, difficulty swallowing, stomach pain, nausea, vomiting and problems in the passage of food or any combination of these symptoms. Semi-structured interview schedules were used for data collection. Standardized tools, previously validated in the Indian settings were also used to collect data. The tools were translated in Hindi language, if not already available in Hindi. Height was measured using stadiometer and weight through electronic weighing scale of the clinic, using standard procedures. Data were collected by the investigators as exit interviews of the patients, after they had consulted the physician of the clinic. The physicians posted in the clinic were briefed about the study, including when to refer the patients to the data collectors. For the diagnosis of GERD, Gastroesophageal Reflux Disease Questionnaire (GERD-Q), a 6-item, questionnaire was used [13]. The participants were asked to reflect on symptoms over the preceding week. For positive predictors, scores from 0 to 3 were applied and for negative predictors, 3 to 0 (reversed order, where 3=none) were applied, the total score ranging from 0 to 18. Based on the level of optimal balance between sensitivity and specificity, cut-off value ≥ 8, GERD was used to diagnose GERD [14]. Previously, in a study in south India, this tool has been successfully used [15]. GERD-Q is free to use without requirement of any permission. For screening for symptoms of anxiety, the Generalized Anxiety Disorder 7-item (GAD-7) scale was used, which has been previously validated for screening of anxiety symptoms in India [16]. For mild, moderate and severe anxiety, scores of 5, 10 and 15 were taken as the cut-off points, respectively. When used as a screening tool, further evaluation is recommended when the score is 10 or greater. Using the threshold score of 10, the GAD-7 has a sensitivity of 89% and a specificity of 82% for GAD. Previous literature reports good reliability and validity of GAD-7 for measuring anxiety in the general population [17]. Patients having GAD-7 score ≥ 10 were referred to the department of Psychiatry of the same hospital for further evaluation. Kobo Toolbox (Harvard Humanitarian Initiative) based electronic form was used for data collection. Data were analysed using IBM SPSS Statistics software. Using Chi-square test was used for testing association between categorical variables. Univariable and multivariable logistic regression analyses were performed to find out the predictors of moderate to severe levels of anxiety (score ≥ 10 in GAD-7). The variables with p-value < 0.25 in univariable analysis were entered in the multivariable model. Unadjusted and adjusted odds ratios were reported for the variables. Statistical significance was set at the p-value of 0.05.

## Results:

A total of 500 participants were recruited in the study. Table 1 (see PDF) provides the details of socio-demographic and clinical characteristics of the study participants. Out of 500, 83 (16.6%) participants had minimal anxiety, 80 (16%) had mild anxiety, 267 (53.4%) had moderate anxiety and 70 (14%) had severe anxiety as per GAD-7 scoring criteria. Therefore, the proportion of participants having anxiety was 83.4% (417 out of 500). Also, 337 participants (67.4 %) suffered from moderate to severe anxiety (according to categorization of scoring for GAD-7 questionnaire *i.e.* had a score ≥ 10) as depicted in the bar chart (Figure 1 - see PDF). The result of univariable logistic regression analysis is given in Table 2 (see PDF). Table 3 (see PDF) shows the results of multivariable logistic regression *i.e.* the factors independently predicting higher severity of anxiety (moderate to severe according to GAD-7 Questionnaire *i.e.* score ≥10). The results showed that participants aged 65 and above had more than 6 times greater odds of suffering from moderate to severe anxiety, as compared to other age groups. The results showed that married participants had 2.33 times the higher odds of suffering from moderate to severe anxiety as compared to their unmarried, divorced, separated or widowed counterparts. Also employed participants had nearly four times higher odds (OR 3.82) of suffering from higher severity of anxiety compared to those who were unemployed. Participants consuming vegetarian diet had 1.68 times higher odds of suffering from moderate to severe anxiety compared to those consuming non vegetarian diet (p value = 0.028*). Participants having high likelihood of GERD according to GERD-Q scale (GERD-Q score ≥8) had close to 3.5 times the odds of suffering from moderate to severe anxiety as compared to those having lower likelihood of GERD (p value<0.001). Participants of upper and upper middle socio-economic status had almost one-fifth odds of having moderate to severe depression as compared to those having middle, lower middle and lower socioeconomic status (OR 0.19, p value = 0.014). Participants who consumed alcohol had almost one third the odds of suffering from moderate to severe anxiety as compared to those who did not consume alcohol (OR 0.29, p<0.001).

## Discussion:

In the present study, we found that 83.4% of the patients having upper GI symptoms screened positive for anxiety symptoms. Multiple studies conducted in the past depict a strong link between upper gastrointestinal symptoms and anxiety [18, 19-20]. Also, increased number of GI symptoms is significantly associated with anxiety symptoms and those who perceived more severe GI symptoms also tend to have a higher incidence of anxiety symptoms [21]. A study conducted in 15 primary care clinics found a significantly increased risk for psychological disorders such as generalized anxiety disorder and panic disorder as the number of GI symptoms increased, specifically, in comparison with patients with no GI symptoms, the risk for anxiety in patients with one, two and three GI symptoms increased 3.7-fold, 6.5-fold and 7.2-fold, respectively [22]. Another study showed that having gastrointestinal problems lead to increase in anxiety by 8.8% [23]. We found that participants who were married and those currently employed were 2.3 and 3.8 times more likely to have anxiety than unmarried and unemployed participants respectively. India's national mental health survey conducted in 2015-2016 showed that 77% of married individuals suffered from anxiety disorders compared to unmarried individual [24]. We could not find any study which has shown positive association between married individuals having upper gastrointestinal symptoms and anxiety. Recent studies done to study the association between marriage and anxiety disorders either does not show any significant association, [25 and 26] or show negative association between marriage and anxiety. A study done in 2022 on Spanish population to see the effects of gastrointestinal disturbances on the onset of depression and anxiety taking data on sociodemographic factors at the national and autonomous community levels depicted that probability of having both anxiety and a gastrointestinal problem decreases by 4.6% with respect to married individuals than others who are not married [23]. A study done by Choi *et al.* in China also showed that the percentage of married subjects showing reflux symptoms in erosive reflux disease group (OR = 0.39) and non-erosive reflux disease group (OR= 0.57) was significantly lower than in the control group (P < 0.001) [22]. These findings are in contrast to our study. This finding could be due to external and environmental factors that affect married individuals, such as stress of employment. Suffering from upper GI symptoms can decrease work productivity, resulting in higher anxiety among the employed. Also, as our data collection continued during the COVID-19 pandemic, the large unemployment wave that affected individuals also posed threat to employed individuals about the future regarding job security, which could have increased the anxiety in married participants, who were also employed [27]. We found increasing anxiety in patients suffering from upper gastrointestinal symptoms with increasing age. The highest association was in the age group of 65 and above which was 6 times higher (OR=6.23). A similar study showed that the probability of having anxiety increases by 0.1% with every one-year increase in age [23]. We found that patients with upper gastrointestinal symptoms with anxiety were more likely to have a vegetarian diet. Although multiple studies showed that consuming a healthy vegetarian diet rich in fibers, legumes nuts, green leafy vegetables and fruits provides essential vitamins such as C and E and minerals such as magnesium which play a pivotal role as antioxidants and thus, prevent the oxidative stress induced damage to neurons in the brain [28, 29- 30], ultimately preventing stress and anxiety development. However, it was also reported that animal derived nutrients also play a pivotal role in preventing anxiety, which is deficient in vegetarians. Neural processes including neuroimmunomodulation, myelination and neurotransmitter synthesis and transmission require nutrients including vitamin D, zinc, iron [31] and omega-3 PUFA, which are typically consumed at lower levels in a vegetarian diet, to coordinate and improve overall brain function. Similarly, a deficiency in vitamin B12 and folate can increase the concentration of homocysteine, an amino acid implicated in neurotoxicity and the pathophysiology of depression and anxiety [32]. Thus, it seems to be a balanced interplay of multiple dietary supplements that play a role in modulating mental illnesses such as stress and anxiety disorders. We found significant protective effect of alcohol on anxiety (OR=0.29). Alcohol has been proven as a risk factor for GERD symptoms and GERD [33], as its consumption reduces the lower esophageal sphincter pressure, facilitating reflux. However, the largest studies have not identified any association between alcohol consumption and GERD [34, 35]. In the largest study available, a nested case-cohort study from Norway with 43,363 participants, more than ten occasions with alcohol consumption during the last two weeks were not associated with any increased risk of developing GERD, adjusted OR (1.0) and there was no dose-response association with increasing consumption (p for trend 0.54) [35]. In a Swedish case-control study of 27,717 twins, increasing alcohol intake even indicated a dose-dependent decreased risk of GERD in the monozygotic co-twin control comparison among women, with adjusted OR of 0.31 (95% CI 0.11 to 0.84) with an alcohol intake of more than 2400 grams per month [36]. However, when the consumption of beer, wine and spirits was analyzed separately, no association with GERD was evident. This suggests that chance might be an explanation of the inverse association. Finally, we found 3.5 times increased likelihood of anxiety in patients having high likelihood of GERD, which was reported in many previous studies. The most plausible reason behind this is the intricate connection of Gut - brain axis. Clinical and preclinical studies have implicated multiple mechanisms in the bidirectional pathways between certain psychiatric disorders and the brain-gut axis including the activation of immune response, the dysfunction of HPA and altered neurotransmitter or neuromodulators levels [37, 38- 39]. Various psychotic symptoms affect the intestinal microbiome through the hypothalamus-pituitary-adrenal gland axis. Conversely, the intestinal microbiome regulates the gastrointestinal tract environment and affects psychological factors by means of the microorganisms or their metabolites, either acting directly on the brain or through the synthesis of various neurotransmitters [39, 40]. Immune cells can produce various neurotransmitters and other factors, which alert the brain to the changes that occur in the body and affect the plasticity of the local and central nervous systems, thereby can regulate mood and behavior [41]. A study has also explored the link of neurotransmitters such as serotonin, tryptophan and indole derivatives which are metabolized by gut microbiota in relation to gut health [42]. Despite high prevalence of both upper gastrointestinal symptoms and anxiety disorders in India, there is a paucity of such studies depicting association between these two diseases. This is one of the main strengths of the present study. There are some limitations as well. As it was a cross-sectional study, there is a possibility of reverse causality. We did not analyse the number of upper GI symptoms and its association with the risk of anxiety and its severity to be able to comment on the clustering effect. As data collection was continued during COVID-19 pandemic also, selection bias may have been there. Collaborative care models integrating several specialty physicians such as primary care physicians, general practitioners, gastroenterologists and mental health professionals should be implemented, focused on screening of symptoms of anxiety among patients reporting with upper GI symptoms, referring those with moderate to severe anxiety to mental health professions for appropriate management, which should be done keeping in mind the predictors of higher severity of anxiety. To detect psychiatric morbidity in medically ill patients, recently some useful tools tested in Indian settings have also been developed. The Hospital Mental Health Screen (HMHS) is one such tool for use in hospital settings [43].

## Conclusion:

We found that 83.4% of the participants had anxiety and 67.4% had moderate to severe anxiety. Age ≥ 65, being married, employed, vegetarian and having high likelihood of GERD were predictors of moderate to severe anxiety, whereas, higher socio-economic status and alcohol consumption were the protective factors. Collaborative care models focused on targeted screening for higher severity of anxiety among adult patients having upper GI symptoms based on the predictors, their referral and appropriate management is the need of the hour.

## Declaration:

It is hereby being declared that no pre-PRINT of this manuscript is published elsewhere.
